# The effect of cerium dioxide nanoparticles on the viability of hippocampal neurons in Alzheimer’s disease modeling

**DOI:** 10.3389/fncel.2023.1131168

**Published:** 2023-03-16

**Authors:** Vita V. Hanzha, Nataliia M. Rozumna, Yevheniia V. Kravenska, Mykola Ya. Spivak, Elena A. Lukyanetz

**Affiliations:** ^1^Department of Biophysics of Ion Channels, Bogomoletz Institute of Physiology, The National Academy of Sciences of Ukraine (NASU), Kyiv, Ukraine; ^2^Danylo Zabolotny Institute of Microbiology and Virology, The National Academy of Sciences of Ukraine (NASU), Kyiv, Ukraine

**Keywords:** hippocampus, cell culture, cerium nanoparticles, beta-amyloid, Alzheimer’s disease, nanomaterials

## Abstract

The possibilities of using nanoparticle materials based on cerium dioxide (CNPs) are exciting since they are low toxic and have specific redox, antiradical properties. It can be supposed that CNPs’ biomedical use is also relevant in neurodegenerative diseases, especially Alzheimer’s disease (AD). AD is known as the pathologies leading to progressive dementia in the elderly. The factor that provokes nerve cell death and cognitive impairment in AD is the pathological accumulation of beta-amyloid peptide (Aβ) in the brain tissue. In our studies, we examined the impact of Aβ 1-42 on neuronal death and evaluated the potential neuroprotective properties of CNPs during AD modeling in cell culture. Our findings show that, under AD modeling conditions, the number of necrotic neurons increased from 9.4% in the control to 42.7% when Aβ 1-42 was used. In contrast, CNPs alone showed low toxicity, with no significant increase in the number of necrotic cells compared to control conditions. We further explored the potential of CNPs as a neuroprotective agent against Aβ-induced neuronal death. We found that introducing CNPs 24 h after Aβ 1-42 incubation or prophylactically incubating hippocampal cells with CNPs 24 h before amyloid administration significantly reduced the percentage of necrotic cells to 17.8 and 13.3%, respectively. Our results suggest that CNPs in the cultural media can significantly reduce the number of dead hippocampal neurons in the presence of Aβ, highlighting their neuroprotective properties. These findings suggest that CNPs may hold promise for developing new treatments for AD based on their neuroprotective properties.

## Introduction

In recent years, new possibilities for the use of nanomaterials with several unique properties in medicine are actively studied to diagnose and treat diseases or improve the human’s physiological functions. Nanomaterials have emerged as promising candidates for neuroprotection due to their unique properties, including small size, high surface area-to-volume ratio, and the ability to penetrate biological membranes. These properties enable nanomaterials to interact with cellular components, such as enzymes and receptors, and modulate cellular signaling pathways. As a result, nanomaterials can exhibit neuroprotective effects by reducing oxidative stress, inflammation, and apoptosis. The possibilities of using nanoparticle materials based on cerium dioxide (CNPs) are especially widely studied (Lord et al., 2021; Saifi et al., 2021).

Cerium dioxide (CNPs) are a type of nanomaterial that has gained significant attention due to their unique redox properties (Yadav, 2022). CNPs can act as an antioxidant and a pro-oxidant depending on the cellular environment. Under oxidative stress conditions, CNPs can act as an antioxidant by scavenging free radicals and reducing oxidative damage. Conversely, under reducing conditions, CNPs can act as a pro-oxidant by generating reactive oxygen species (ROS) and inducing apoptosis in cancer cells (see for review Chen et al., 2013). Their characteristics, such as low toxicity and specific redox and antiradical properties, the ability to regenerate, allow to consider CNPs as a promising object for biomedical applications (Celardo et al., 2011). For example, CNPs has been shown to reduce retinal degeneration (Wong and McGinnis, 2014), protect from oxidative stress brain tissue (Heckman et al., 2013), improve outcome after mild traumatic brain Injury (Bailey et al., 2020), human skin fibroblasts (Lee et al., 2013), endothelial cells (Chen and Stephen Inbaraj, 2018), and heart (Niu et al., 2007).

It seems, CNP’s biomedical use is also relevant in neurodegenerative diseases, especially Alzheimer’s disease (AD) (Singh et al., 2008; D’Angelo et al., 2009; Naz et al., 2017; Zand et al., 2019). It is known that AD is one of the pathologies that leads to progressive dementia in the elderly. The hippocampus is the first area of the brain damaged by senile plaques at the course of AD. Therefore, the problems with memory, speech, and disorientation appear among the main neurobehavioral symptoms at AD (Alzheimer’s Disease International [ADI], 2019). It is known that beta-amyloid peptide (Aβ) is a major component of the plaques–characteristic morphological features of AD (Haass and Selkoe, 2007). According to the “amyloid cascade” hypothesis, the factor that leads to nerve cell death and cognitive impairment is the pathological accumulation of Aβ aggregates in the brain tissue. Neurotoxicity of Aβ is manifested by impaired Ca^2+^ homeostasis, mitochondrial dysfunctions (Kravenska et al., 2016, 2020), induction of oxidative stress, excitotoxicity, inflammatory processes, intensification of apoptosis, and necrosis (Marchesi, 2011; Kravenska et al., 2016). In the context of neuroprotection, CNPs have been shown to protect neurons from oxidative stress-induced cell death. Specifically, CNPs can scavenge ROS and restore the redox balance in neurons. Additionally, CNPs can upregulate antioxidant enzymes and reduce inflammation in the brain. These mechanisms can protect neurons from neurodegeneration and promote their survival. In AD, CNPs have been investigated as a potential therapeutic agent due to their neuroprotective effects (Danish et al., 2022). CNPs have been shown to reduce Aβ-induced toxicity *in vitro* and *in vivo* by reducing oxidative stress, inflammation, and Aβ aggregation. Additionally, CNPs can penetrate the blood-brain barrier and accumulate in the brain, making them a potential candidate for delivering therapeutics to the brain (see for review Stephen Inbaraj and Chen, 2020).

In conclusion, CNPs possess unique properties that make them promising candidates for neuroprotection, especially in the context of AD. The mechanism of neuroprotection by CNPs involves scavenging ROS, restoring redox balance, and reducing inflammation in neurons. Further research is necessary to fully understand the potential of CNPs as a therapeutic agent for AD and other neurodegenerative diseases.

Our investigations aim to test the effects of CNPs on hippocampal neurons’ viability during modeling AD on cell cultures.

## Materials and methods

### Animals

All experimental procedures followed the European Commission Directive (86/609/EEC) and ethical guidelines of the International Association for the Study of Pain and were approved by the local Animal Ethics Committee of the Bogomoletz Institute of Physiology (Kyiv, Ukraine). All efforts were made to minimize the number and suffering of animals used.

### Preparation of primary dissociated hippocampal cell culture

Studies were performed on rat hippocampal culture neurons using a common technique described earlier (Lukyanetz et al., 2003a,b). To do this, a total of 12 newborn Wistar rats were used. They were decapitated, the hippocampus was isolated in a sterile and cold Petri dish, cut into several pieces each, and transferred for 10 min to a warm (36°C) enzyme solution containing 0.25% trypsin (Sigma-Aldrich, St. Louis, MO, USA). Next, the pieces of the hippocampus were washed several times with cold nutrient medium. The tissue was dispersed to homogeneous suspension using a 1 ml tip of pipetter. Using a Goryaev chamber and adding the required volume of nutrient medium, a suspension with a density of 3 × 10^5^ cells per 1 ml was prepared. Then 200 μL of the cell suspension was applied to 20 mm × 20 mm slides, which were pre-treated with polylysine (0.05 mg/ml, Sigma-Aldrich) and laminin (0.005 mg/ml, Sigma-Aldrich). After 2 h incubation at 37°C in an atmosphere enriched with 5% CO_2_, in each Petri dish was added 2 ml of nutrient medium, which included 90% of the minimum essential media (MEM, Sigma-Aldrich), 2.2 g/l NaHCO_3_, 10% horse serum (Gibco, Cat. No. 16050130, Auckland, New Zealand), 10 μg/ml insulin and antibiotics: 50 IU/ml benzylpenicillin sodium and 50 μg/ml streptomycin sulfate. Neurons were cultured in an incubator for 2 weeks at 37°C in an atmosphere enriched with 5% CO_2_. To inhibit glial cells’ proliferation after 3 days *in vitro* culture was treated with 1 μM/L cytosine arabinoside (AraC) (Sigma-Aldrich) for 24 h. After that, a complete replacement of the nutrient medium of neurons was performed. Every 4 days, 400 μL of the solution of cultivating medium from the Petri dish was replaced with a fresh one. Neurons were taken for the experiment on 11–13 days of cultivation (see Supplementary Figure 1).

### Treatment of hippocampal cells with reagents. Modeling of neurodegeneration

Prolonged culturing of the central nervous system (CNS) cells *in vitro* is widely used as the AD study model (Kravenska et al., 2016; Bailey et al., 2020). In our studies, the AD model was obtained by 24 h incubation of hippocampal culture neurons with Aβ1–42-amyloid (Sigma-Aldrich, USA) at a final concentration of 2 μM. A separate group of cell cultures was co-incubated with Aβ1-42 (2 μM, 24 h) and CNPs (1: 100 dilution of 0, 1 mM stock) 24 h in different configurations of administration of active substances to study the effect of CNPs on the viability of neurons in this model of AD (see Supplementary Figure 1C). CNPs were obtained by the chemical method described previously in detail (Rybalko et al., 2021) from the Institute of Microbiology and Virology of NAS of Ukraine, Kyiv, Ukraine. The synthesis method enabled the preparation of cerium samples with crystallite sizes of about 3 nm. X-ray diffraction (XRD) has shown that the used samples are single-phase and correspond to cubic CeO_2_. A concentrated solution of Aβ1–42-amyloid and CNPs was prepared on dimethyl sulfoxide (DMSO) and stored at –20°C. The final concentration of dimethyl sulfoxide did not exceed 0.5%. The study of the possible effect of the DMSO solvent itself on culture cells of the hippocampus of rats was carried out earlier by us. It was shown that its concentrations of 0.5% and less, when added to the culture medium for 24–48 h, did not affect the parameters of the cells in the culture and did not differ from the control group.

### Detection of different types of cell death

**FIGURE 1:**
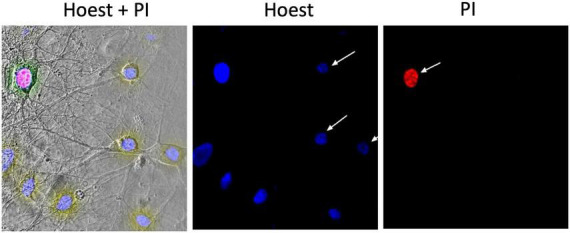
Example of a micrograph of cultured hippocampal neurons. The **(left)** part shows a phase-contrast image merged with images presented to the **(right)**; image in the **(middle)** demonstrates the fluorescence of Hoechst 33258 in neurons; **(right)** the fluorescence of propidium iodide (PI) in the necrotized neuron.

The number of alive, cytologically normal cells and cells with manifestations of apoptosis or necrosis was evaluated using the double staining with dyes Hoechst 33258 (Sigma-Aldrich) and propidium iodide (PI, Sigma-Aldrich), Figure 1 (left part). The first of these dyes penetrate undamaged cell membranes and stains nuclear chromatin, thus providing visualization of cytologically normal and apoptotically altered cells, which have so far remained viable (Figure 1). In alive cells, chromatin is distributed more evenly throughout the nucleus’ volume, and Hoechst 33258 in them fluoresces faintly with blue light. In apoptotic cells, Hoechst 33258 intensity of fluorescence 3–4 times higher than in normal cells (bright blue glow), indicating condensation of chromatin and fragmentation of nuclei, which occurs during the induction of apoptosis (Figure 1, central image). PI cannot penetrate the intact plasma membrane and stains only the nuclei in cells with a significantly damaged plasmalemma, i.e., cells in which occurred necrotic transformation–necrosis (red color fluoresce) (Figure 1, right part).

A protocol was used according to the previously described technique to stain the neurons of hippocampal culture (Kravenska et al., 2016). Coverslips with control neurons or cultures treated with amyloid and/or CNPs in different configurations of administration of active substances were transferred for 10 min in a PI (final concentration 2.0 μg/ml). The cells were then fixed by immersion for 20 min in a 4% solution of paraformaldehyde, then placed for 20 min in Hoechst 33258 solution with a final concentration of 1.0 μg/ml. After each staining step, the cells were washed twice in 2.0 ml of phosphate buffer (0.1 M). All manipulations were performed at room temperature. After staining, the coverslips with cells were mounted on slides using a solution of Aqua-Poly Mount (Polysciences, Inc., Warrington, PA, USA). Fixed and mounted cells were studied using confocal microscopy.

### Confocal laser scanning microscopy

The FV1000-BX61WI confocal laser scanning microscope and the software FluoView (Olympus, Japan) or Image J (National Institutes of Health, Bethesda, MD, USA) were used in the studies to obtain fluorescent images and count of neurons. The laser excitation wavelengths were 352/405 nm for Hoechst 33258 and 543 nm for PI, respectively.

Neurons were distinguished from glial cells by phase-contrast and were counted in five spatially distant areas of each sample. Each sample contained 100 to 300 cells, the number of living, apoptotic, and necrotic cells were counted. The values of every type normalized to the total cell number of the sample, and then the values of all samples were averaged for each type of cell state.

### Statistical analysis

The obtained results were processed by methods of variation statistics using the program Origin 7.0 (OriginLab Corporation, Northampton, MA, USA). Numerical data are given as means ± mean error. A normal distribution characterized the results, intergroup comparison of data was performed using ANOVA analysis of variance. If intergroup differences were found, the Tukey test was used. The results were considered statistically significant at *P* < 0.05.

## Results

Our research was devoted to establishing a possible neuroprotective role of CNPs on the viability of hippocampal culture neurons in the simulation of AD. To this end, five groups of experiments were conducted (see Supplementary Figure 1): the first—cells were cultured in control conditions; the second–the introduction of CNPs in cultural media for 24 h before the measurement; third–the introduction of amyloid Aβ1-42 in media for 24 h before the measurement; fourth–the introduction of amyloid Aβ1-42 for 24 h, and the subsequent introduction of CNPs for 24 h before the measurement; fifth–prophylactic administration of CNPs into media for 24 h before the introduction of amyloid Aβ1-42. The cells were then dual stained with two DNA-binding dyes (Hoechst and PI), and then they were investigated and counted using confocal laser scanning microscopy. The incubation duration of hippocampal cultures in all studied groups was the same. On the 11th and 12th days of cultivation, reagents were added, and on the 13th day, the samples of all 5 groups of experiments were stained and fixed (see Supplementary Figure 1C). The study groups were carried out in quadruplicate.

**FIGURE 2:**
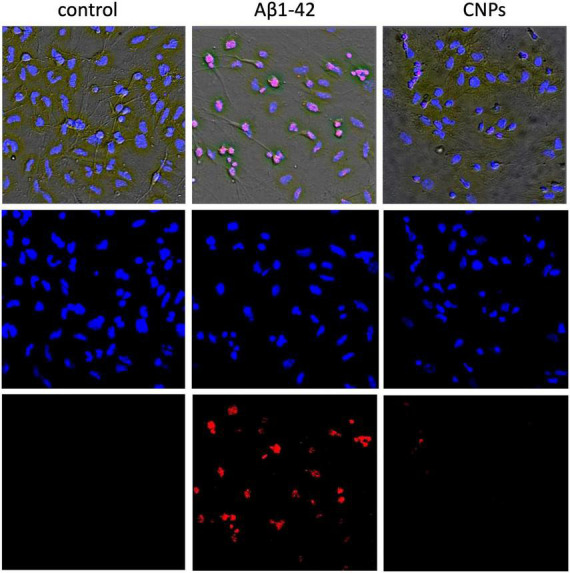
Micrographs of cultured hippocampal neurons after Aβ or CNPs incubation. The **(left)** column shows images of cells in control conditions. The next column demonstrates cells incubated with Aβ1-42 (24 h). The **(right)** column shows cells after incubation with CNPs (24 h). Above are phase-contrast images, merged with images obtained by staining with Hoechst 33258 (below in the column) and propidium iodide (PI) (lowest).

In control samples of rat hippocampal cell culture, the vast majority of cells (average 84.7 ± 2.1%) did not show any pathological changes (Figure 2, left column). Figure 2 shows merged images: phase-contrast, Hoechst, and PI recorded (upper), and separately showed for Hoechst (below) and PI (lowest) stained images. The nuclei of such cells, which accumulated the Hoechst 33258, were characterized by weak blue fluorescence and had clear contours, nuclear chromatin was stained relatively evenly (Figure 2). The nuclei of a relatively small part of the neurons (5.9 ± 1.31%) in the control samples gave bright blue fluorescence. They had a pronounced fragmentation of chromatin, which was a sign of apoptotic transformation. There was also a portion of neurons in control samples (9.4 ± 1.57% on average in the group), stained with PI. They were characterized by red fluorescence, which indicated necrotic degeneration of this part of the analyzed neuronal populations. The presence of several dead cells in the control conditions is explained by their damage during culture preparation, particularly in the process of changing the culture medium.

In the second series of experiments, cells were treated with amyloid β1–42. After incubation of hippocampal cell culture samples in amyloid β1–42 medium, cytologically normal cells accounted for approximately one-third of the study population (mean 31.8 ± 3.02%). Neurons with apoptotic changes in these conditions were observed approximately five times more often than in control (25.5 ± 4.32%) (Figure 2, middle column). The group of cells with necrosis signs was also more numerous than in control samples (on average, their number was 42.7 ± 4.17%). Thus, amyloid β1–42 in the culture medium induced intensive death of hippocampal neurons: more than half of the cells of the studied sample showed pronounced pathological changes that developed in both apoptotic and necrotic scenarios.

In the third part, the introduction of CNPs showed that the proportion of living neurons averaged 81.1 ± 2.35%, apoptotic–10.1 ± 2.2%, necrotized–8.8 ± 1.34% of the total number of examined cells. Probable changes in groups of cells in comparison with control were not revealed. Therefore CNPs had a low toxic effect on cells of a hippocampus (Figure 2, right column).

**FIGURE 3:**
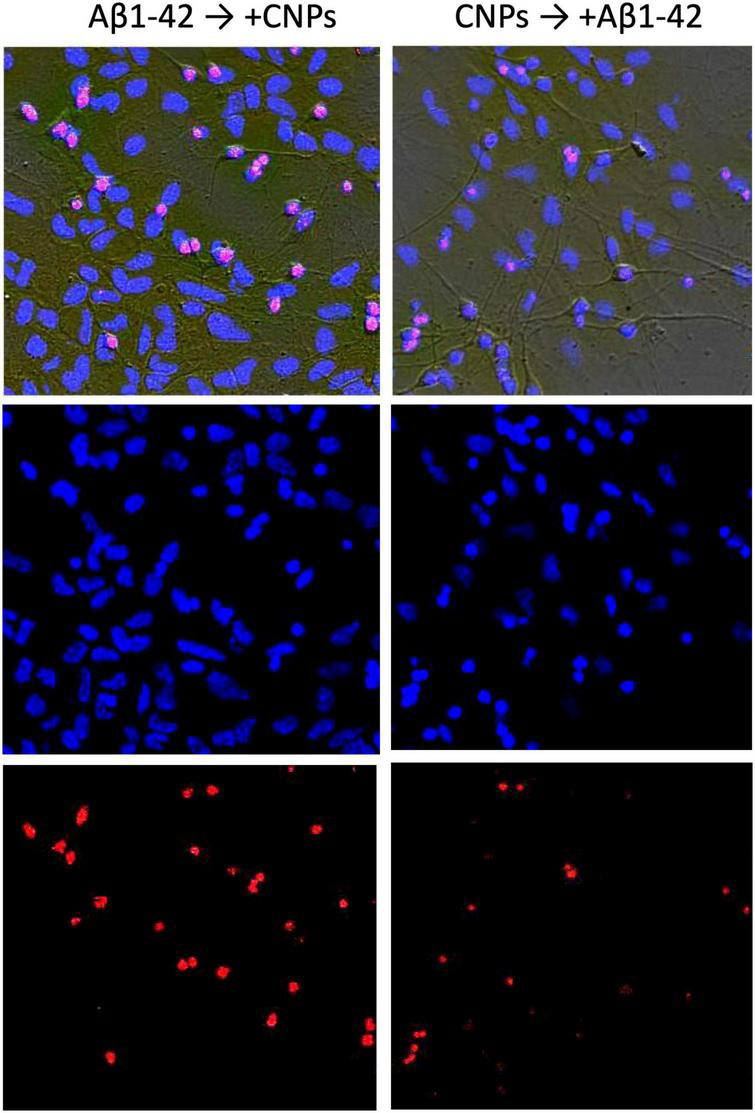
Effect of nanoparticles CNPs on the influence of Aβ on hippocampal neurons. The **(left)** column shows images of cells incubated with Aβ1-42 (24 h) and subsequent adding CNPs for the next 24 h. The **(right)** column demonstrates the images where the cells were incubated with CNPs for 24 h before introducing Aβ1-42 (incubation 24 h). Above in columns are phase-contrast images, merged with images obtained by staining with Hoechst 33258 (below in the column) and propidium iodide (PI) (lowest image in the column).

**FIGURE 4:**
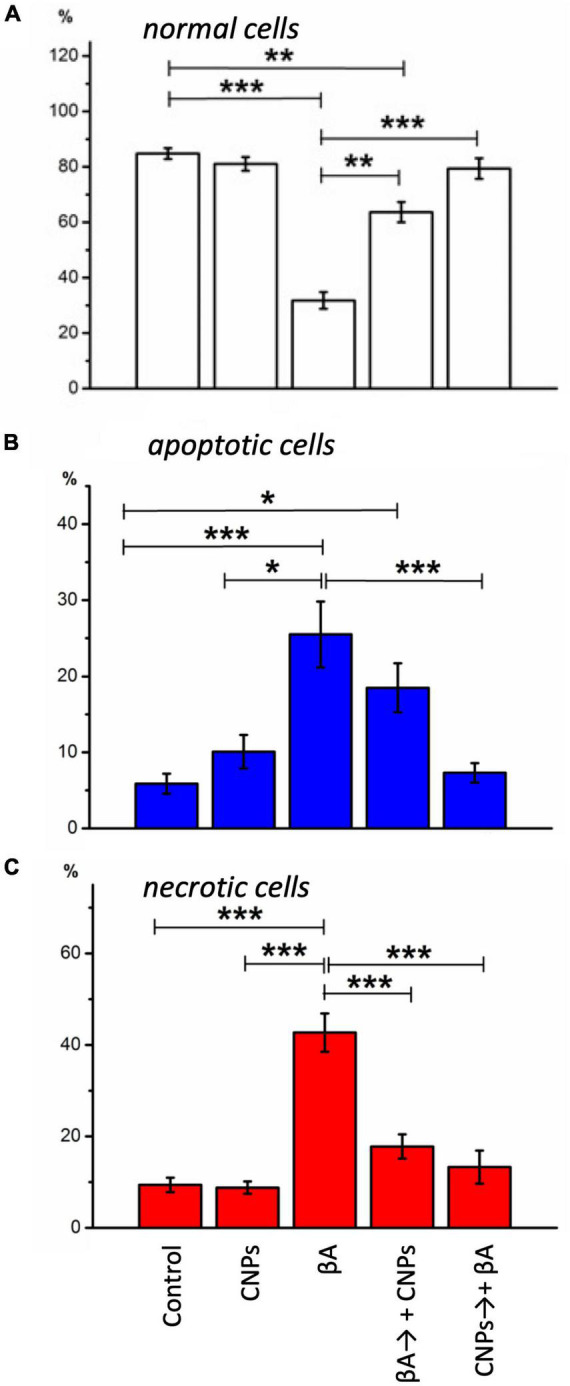
Diagrams of the relative numbers neurons of different states in distinct experimental conditions are shown. **(A)** The healthy cells; **(B)** cells with signs of apoptosis; **(C)** necrotic neurons in cells’ culture are shown. The percentage of each type from the total number of neurons on the glass was calculated. Measurements were made: in control, during incubation with CNPs, with Aβ1-42, during incubation with Aβ1-42, and subsequent adding of CNPs 24 h before the measurement, prophylactic administration CNPs for 24 h before the introduction of Aβ1-42. Vertical scale–normalized values of the number of cells, %. *P < 0.05, **P < 0.01, ***P < 0.001.

The introduction of CNPs after 24 h of incubation with amyloid β1–42 ensured the preservation of a slightly larger number of alive cytological cells than under conditions of action of alone amyloid β1–42 (Figure 3, left column, Figure 4A). The relative number of cells without signs of degeneration, in this case, was more than half of the analyzed sample (average 63.7 ± 3.64%) and differed from both the control and the corresponding in the action of alone amyloid β1-42. The number of cells with apoptotic changes in this group was approximately three times greater than the control conditions. Still, this number was less than the same value in the above group of samples with the action of alone amyloid β1-42. On average, in the group, the relative number of cells with apoptosis signs was 18.5 ± 3.23% of the total (Figure 4B). The addition of CNPs provided a reduction in the number of apoptotic units by an average of 7% compared with that observed in the previous group (*P* < 0.001) (Figure 4B). The relative numbers of cells with pronounced necrotic changes in these two groups also differed approximately 2.5 times (with the introduction of CNPs after 24 h of incubation with amyloid β1-42 in the media averaged 17.8 ± 2.65%). Treatment with CNPs after the action of amyloid β1-42 suspended its pathological effect on the neurons of hippocampal culture.

After prophylactic incubation of hippocampal cells with CNPs 24 h before amyloid administration, the proportion of living neurons in the total number of cells was 79.4 ± 3.71% (approximately 2.5 times more than in the isolated action of amyloid β1-42), apoptotic–7, 3 ± 1.28%, necrotized–13.3 ± 3.61% (approximately three times less than in the isolated action of amyloid β1–42). In other words, pretreatment of cultures of CNPs hippocampal cells dramatically reduced the intensity of death of these cells due to amyloid β1–42 in both apoptotic and necrotic pathways (Figures 4B, C). Thus, in our experiments, we found that pretreatment of CNPs cultures resulted in almost complete preventing of amyloid action, which induces degeneration nerve cells. These nanoparticles also significantly reduced such negative amyloid β1–42 consequences in the case of administration of CNPs after amyloid itself.

## Discussion

It is known that CNPs biological activity is associated with oxygen non-stoichiometry, i.e., with the existence on the surface of nanoparticles of Ce^3+^ ions. Due to the low value of the redox potential of the pair Ce^4+/^Ce^3+^, near-surface cerium ions easily interact with oxygen, change their valence by 4^+^, and then are reduced back to 3^+^(Coelho et al., 2019). This property determines the main value of CeO_2_ nanocrystals for biomedicine–the ability to participate in redox processes taking place in the body (Tarnuzzer et al., 2005). Therefore, observed in our experiments the effect of the nanoparticles CNPs, can be explained by the ability to participate in redox processes in the brain, especially in the inactivation of ROS, including free radicals (Bailey et al., 2020), which are known to be formed under the Aβ influence and lead to apoptosis or cell necrosis. Besides, after a short period, the nanoparticles can regenerate and are again able to perform an antioxidant function.

According to Heckert et al. (2008) works, the mechanism of inactivation of free radicals by cerium dioxide nanoparticles is similar to the action of superoxide dismutase. Studies of CNPs with hydrogen peroxide by X-ray photoelectron spectroscopy and UV spectroscopy have shown an increase in Ce^3+^ ratio: Ce^4+^ in nanoparticles is directly correlated with an increase in their ability to perform the functions of superoxide dismutase. These results convincingly confirm that the most significant factor is Ce^3+^ in the surface layer.

It should be emphasized that today the number of studies similar to ours is quite limited (Singh et al., 2008; Kwon et al., 2016; Nelson et al., 2016). It is generally known that at the early stage of development of pathological phenomena accompanying AD, oxidation processes are disrupted. Excessive generation of ROS under the influence of β-amyloid leads to increased lipid peroxidation. The result is an intensification of apoptosis and necrotic cell death. According to Singh et al. (2008), CNPs exhibit potent antioxidant activity that depends on particle size, composition, and surface area, and can protect brain culture neurons from free radical damage and β-amyloid 1-42 toxicity. Recently, Kwon et al. (2016) highlighted CNPs therapeutic candidacy to reduce oxidative stress in AD. Their study showed that conjugated CNPs were localized predominantly in mitochondria, reducing reactive gliosis and mitochondrial damage in the AD model. These results are, to some extent, coincide with our observations.

Taking into account the obtained results, we can conclude the next: the experiments have shown that the percentage of dead neurons in control and CNPs did not differ significantly, indicating low toxicity of CNPs to the hippocampal neurons. It was found that the introduction of CNPs significantly reduces the number of dead neurons in hippocampal culture with modeled AD. Thus, on the cultural model of AD, CNPs exhibited neuroprotective properties. Thus, the use of CNPs for neurological applications is very promising, such as creating new neuroprotective drugs for the treatment and prevention of neurodegenerative diseases of the brain based on the composition of CNPs. Therefore, the presented research in this brief report was to show the presence of the cerium effect itself. In our further studies, we will investigate the mechanisms of this protective effect of cerium in detail.

## Conclusion

To sum up, our study investigated the potential of cerium dioxide nanoparticles (CNPs) to protect hippocampal neurons against beta-amyloid peptide (Aβ) induced toxicity, which is a hallmark of AD. Our results show that CNPs have low toxicity and are capable of protecting hippocampal neurons from Aβ-induced necrosis. Specifically, we found that introducing CNPs either after Aβ incubation or prophylactically incubating cells with CNPs before Aβ administration significantly reduced the percentage of necrotic cells. These findings suggest that CNPs may have neuroprotective properties that hold promise for developing new treatments for AD.

However, our study has some limitations. First, we used a cell culture model, which may not fully replicate the complexity of AD pathogenesis in the human brain. Second, we focused solely on the neuroprotective properties of CNPs and did not investigate their potential therapeutic effects on cognitive function in AD animal models or humans. Further studies are needed to explore CNPs full therapeutic potential, leading to the development of effective treatments for this devastating disease.

In conclusion, our study provides evidence of the potential of CNPs as a neuroprotective agent against Aβ-induced toxicity in neurons, highlighting their exciting possibilities in developing new treatments for AD. Further studies are needed to explore CNPs’ full therapeutic potential, leading to the development of effective treatments for this devastating disease.

## Data availability statement

The original contributions presented in this study are included in the article/Supplementary material, further inquiries can be directed to the corresponding author.

## Ethics statement

The animal study was reviewed and approved by the Animal Ethics Committee of the Bogomoletz Institute of Physiology (Kyiv, Ukraine).

## Author contributions

NR conducted the literature review and the first draft of manuscript writing and fulfilled confocal imaging experiments and analyses. VH fulfilled cell culturing, chemical treatments, tested amyloid, and fulfilled a stain of cells and making cell samples for fluorescent analysis. YK fulfilled part of confocal imaging experiments and data analyses. MS ensured the availability of nanoparticles and advised their use in experiments. EL fulfilled supervision, project administration, the studies’ conceptualization, created the study design, image processing, contributed to manuscript revisions, and translation. All authors contributed to the article and approved the submitted version.
