# Supplementary material for: The effect of cerium dioxide nanoparticles on the viability of hippocampal neurons in Alzheimer’s disease modeling
Source: Front Cell Neurosci. 2023 Mar 16;17:1131168. doi: 10.3389/fncel.2023.1131168 (PMC10060808; doi:10.3389/fncel.2023.1131168)
Supplement: Supplementary file 1 [file Data_Sheet_1.docx]

Supplementary Material

**The effect of cerium dioxide nanoparticles on the viability of hippocampal neurons in Alzheimer’s disease modeling**

Vita V. Hanzha, Nataliia M. Rozumna, Yevheniia V. Kravenska, Mykola Ya. Spivak and Elena A. Lukyanetz

* Correspondence: Rozumna N.M.: [nata_nr@biph.kiev.ua](mailto:nata_nr@biph.kiev.ua)


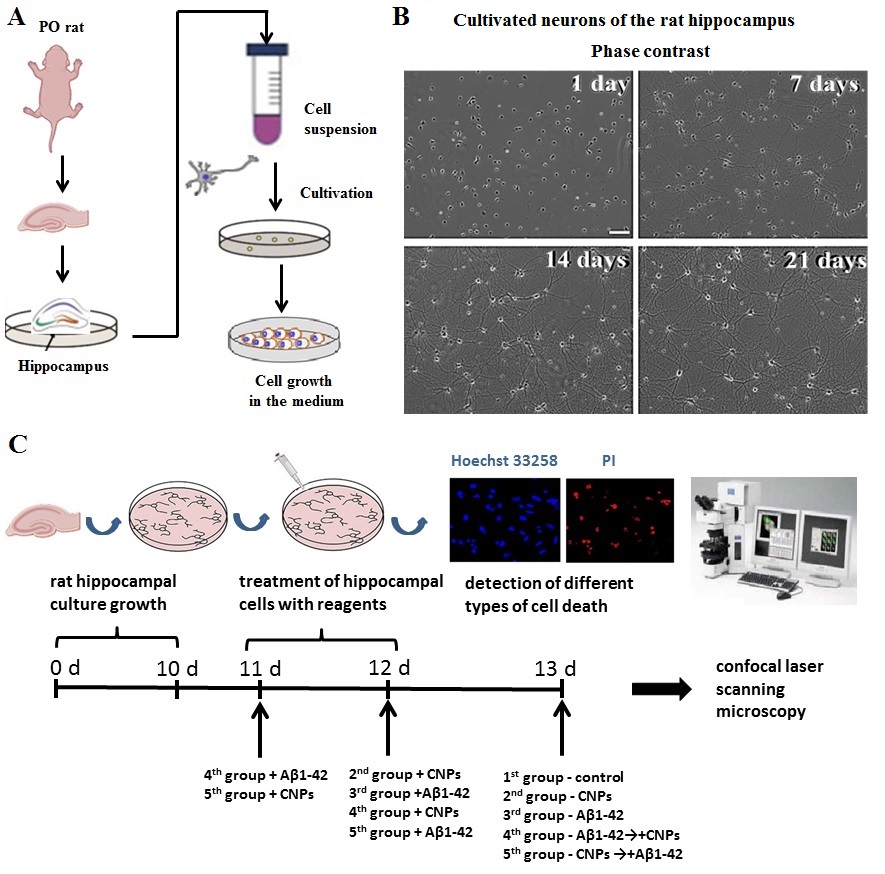


**Supplementary Figure 1.** Design of the experiment. **A**, establishing of primary cultures of rat hippocampal neurons; **B**, examples of phase contrast micrographs of rat hippocampal cultures neurons during cell growth; **C**, schematic representation of the main steps for experiments.
